# Multibacillary Leprosy With Accelerated Progression: A Case Report of an Atypical Clinical Presentation and Therapeutic Approach

**DOI:** 10.7759/cureus.103546

**Published:** 2026-02-13

**Authors:** Carmen Reyes Reyna, David Fernando Narvaez-De Los Rios, Daniel Ibanez Vasquez, Sofia Simaluisa, Anabel Huaman Boza, Alison Cristina Jimenez Ordoñez

**Affiliations:** 1 Internal Medicine, Universidad Nacional Mayor De San Marcos, Lima, PER; 2 Cardiology, Fundación Valle Del Lili, Cali, COL; 3 Internal Medicine, Universidad Privada Del Valle, Cochabamba, BOL; 4 Internal Medicine, Faculty of Health Sciences “Eugenio Espejo,” Universidad Tecnológica Equinoccial, Quito, ECU; 5 Internal Medicine, Universidad Continental, Huancayo, PER; 6 General Medicine, Universidad Latina, San Jose, CRI

**Keywords:** clinical case report, erythema nodosum leprosum, hansen disease, leprosy, migrant health, multibacillary leprosy, multidrug therapy, mycobacterium leprae, neglected tropical diseases, type 2 leprosy reaction

## Abstract

Leprosy, or Hansen’s disease, remains a public health concern, especially in endemic regions and among migrant populations. This case involves a patient from Venezuela living in Peru who initially presented with fever, weight loss, and respiratory symptoms without radiological findings. Physical examination revealed multiple erythematous nodules, hypopigmented macules, and thickened peripheral nerves. A skin smear confirmed multibacillary leprosy.

The patient began standard multidrug therapy and showed gradual improvement. However, by the third month, he developed erythema nodosum leprosum (type 2 reaction), which was successfully treated with thalidomide without interrupting the main regimen. Follow-up showed complete resolution of skin lesions and neurological recovery.

This case highlights an atypical presentation of multibacillary leprosy mimicking a respiratory infection. It emphasizes the importance of clinical suspicion in non-endemic areas with high migration rates. Early diagnosis and treatment are key to preventing permanent complications. Strengthening epidemiological surveillance and comprehensive care are essential for effective control of Hansen’s disease.

## Introduction

Leprosy, or Hansen’s disease, is a chronic infectious condition caused by *Mycobacterium leprae*, an intracellular, gram-positive, acid-fast bacillus with tropism for dermal macrophages and Schwann cells, explaining its characteristic cutaneous and neurological involvement [1]. It remains a public health concern in regions with precarious socioeconomic conditions and is listed among the 20 most impactful neglected tropical diseases affecting vulnerable populations. In 2023, a total of 182815 new cases were reported worldwide, 13.6% of which occurred in the Americas, with Brazil accounting for more than 90% of those cases [2].

Clinical manifestations depend on the host’s cellular immune response, resulting in a spectrum ranging from the tuberculoid form, associated with a competent immune response, to the lepromatous form, characterized by the absence of specific immunity [3,4]. Diagnosis is primarily clinical, supported by slit-skin smear microscopy or histopathological confirmation through skin biopsy [1].

Although curable with multidrug therapy (MDT), delayed diagnosis may result in irreversible nerve damage, deformities, and disability. Additionally, the social stigma associated with leprosy significantly impairs quality of life. Despite therapeutic advances and a global reduction in incidence, leprosy remains a diagnostic challenge in low-endemic regions and should therefore be considered in the differential diagnosis of chronic dermatoneurological syndromes [1].

We report the case of a 31-year-old man residing in Cajamarca, Peru, diagnosed with multibacillary leprosy confirmed by dermal smear microscopy. During treatment with rifampicin, clofazimine, and dapsone, he developed a type 2 reaction (erythema nodosum leprosum (ENL)), which responded favorably to thalidomide. MDT was continued, and progressive clinical improvement with resolution of skin lesions was observed.

## Case presentation

A 31-year-old male, born in Venezuela and residing in Peru for the past five years, working as a carpenter, presented to the emergency department with a fever of 20 days’ duration. His past medical history included a Rickettsia infection successfully treated with doxycycline and azithromycin in 2015. He denied any history of tuberculosis, HIV infection, or other sexually transmitted diseases.

The illness began insidiously with intermittent, non-quantified febrile episodes accompanied by productive cough with yellowish sputum, asthenia, anorexia, and unintentional weight loss of approximately 15 kg. During the respiratory phase, he noticed the appearance of multiple non-tender erythematous nodules on both upper and lower extremities, some of which evolved into superficial ulcers. Hypopigmented macules with sensory loss also appeared on the trunk.

**Figure 1:**
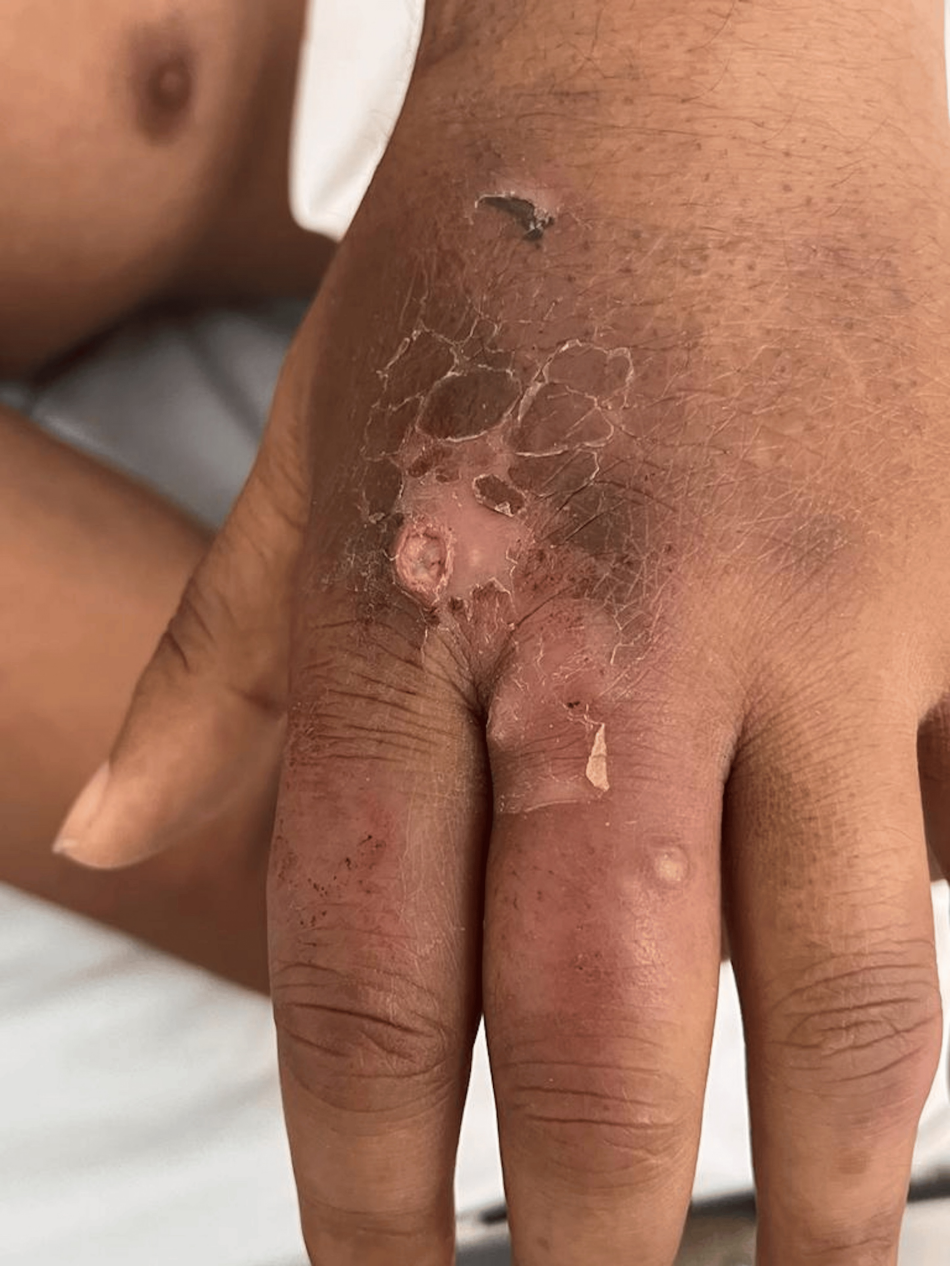
Erythematous and ulcerated nodules on the dorsum of the hand Ulcerated nodular lesion on the dorsum of the hand, showing an erythematous, infiltrated border over a hyperpigmented, thickened base with peripheral scaling and areas of hemorrhagic crust. Mild edema of the dorsal hand and surrounding infiltrated skin is also noted.

**Figure 2:**
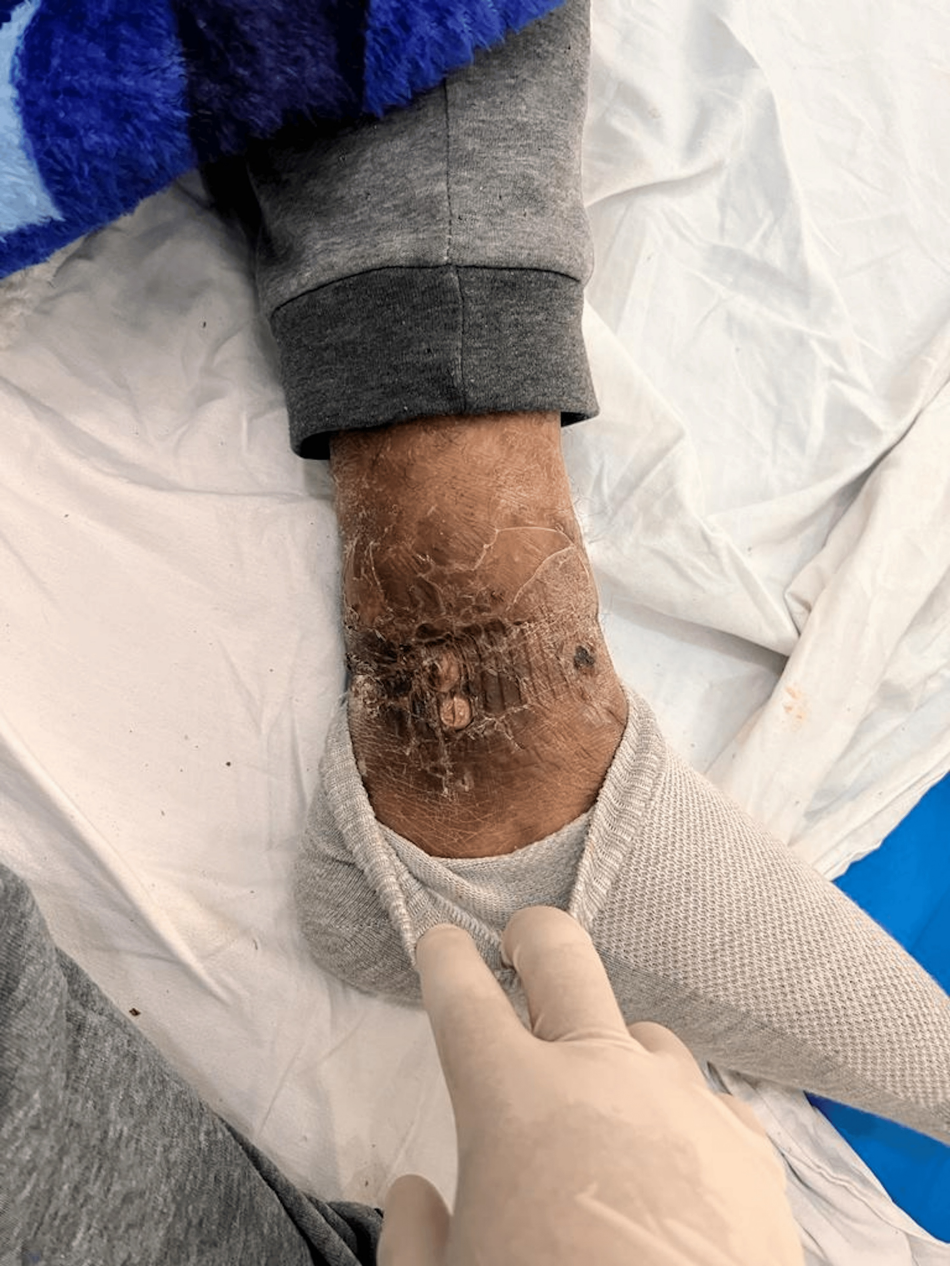
Hyperkeratotic, scaly, erythematous lesions on the right ankle and distal third of the leg, with areas of crusting and fissuring

**Figure 3:**
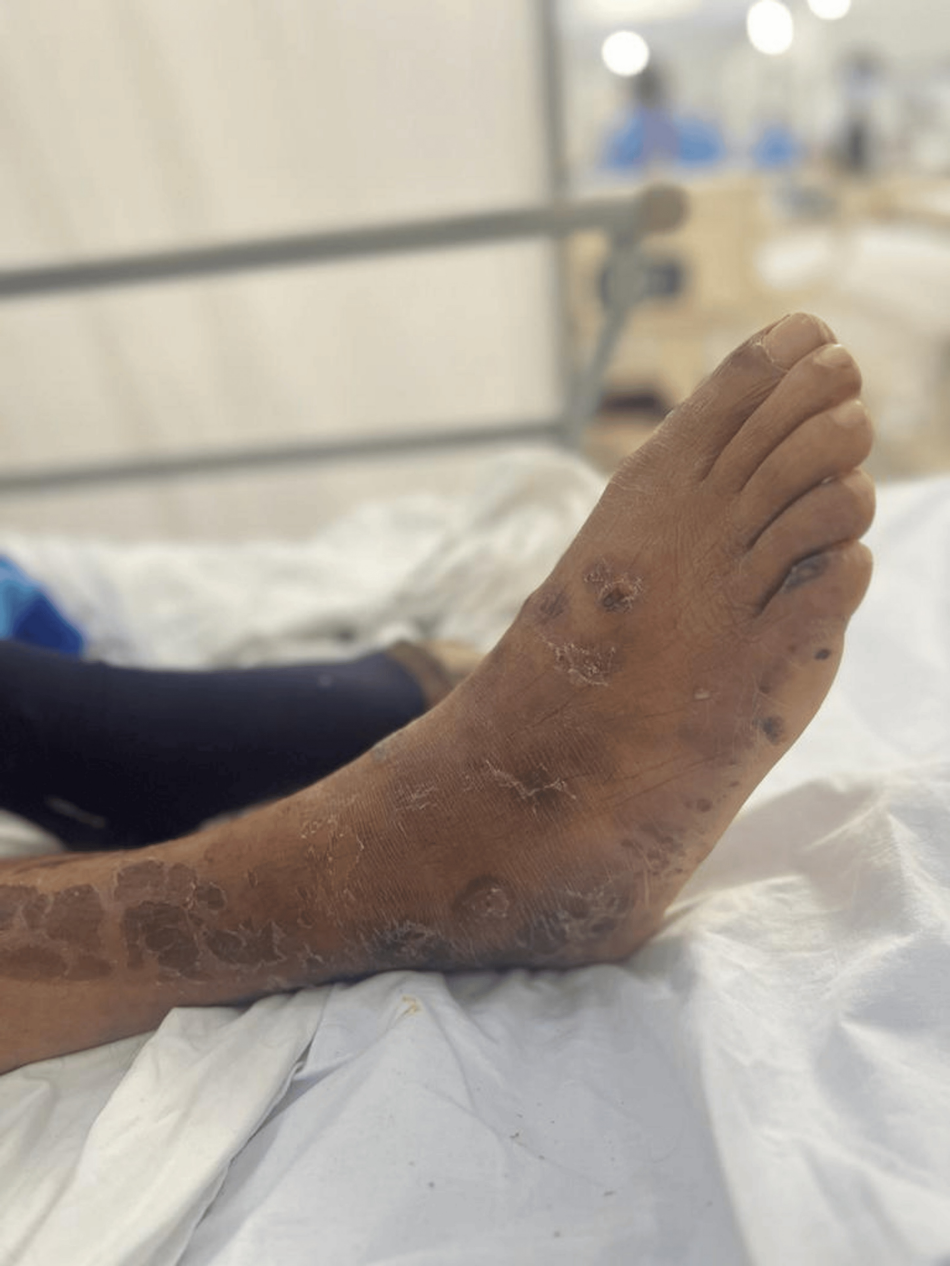
Hyperkeratotic, scaly, erythematous plaques on the lateral aspect of the right foot, with areas of crusting and chronic cutaneous changes

**Figure 4:**
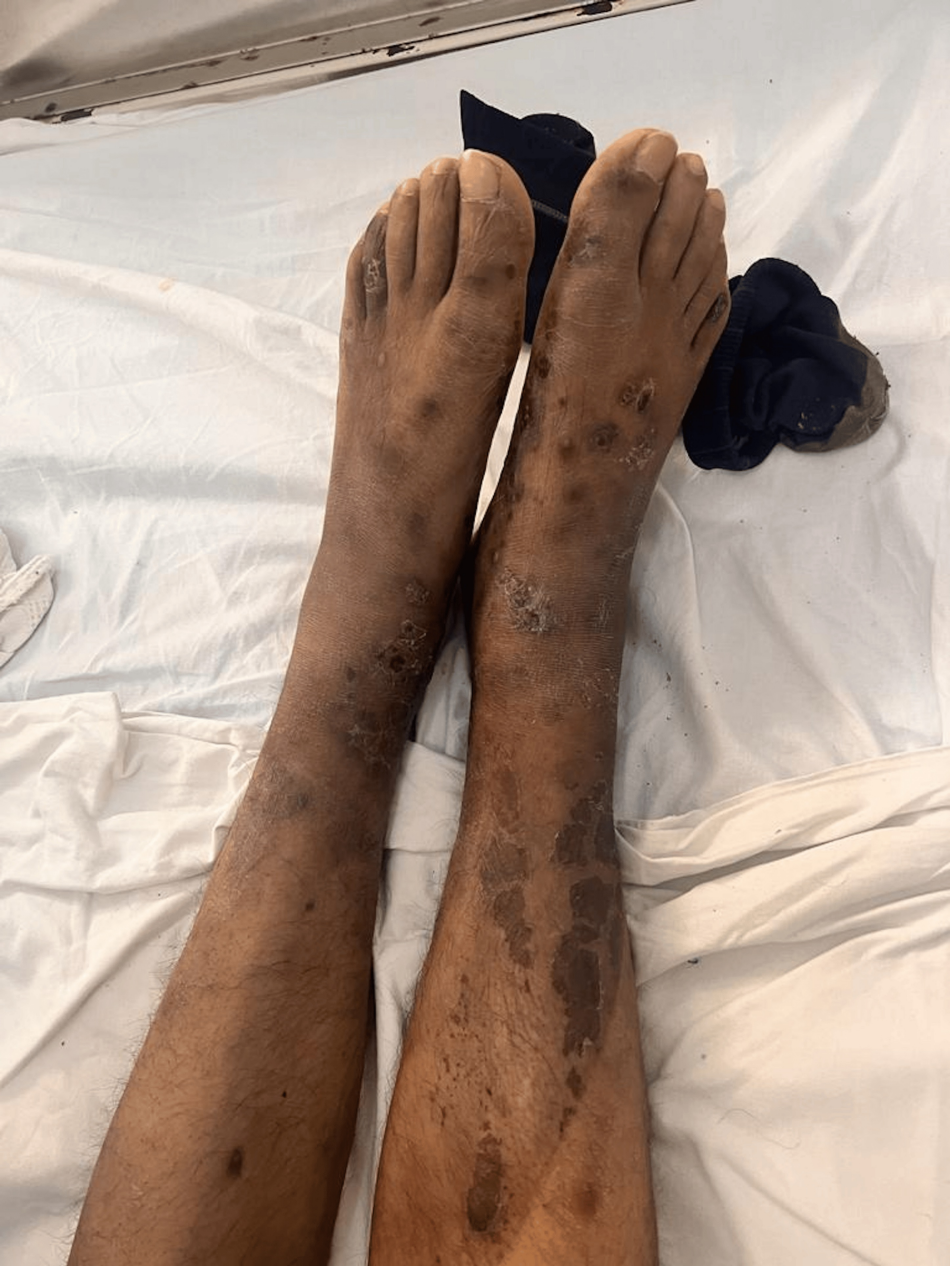
Diffuse hyperpigmented macules and plaques distributed along both lower limbs Diffuse hyperpigmented macules and plaques were distributed along both lower limbs, with signs of chronic cutaneous involvement and irregular skin texture, suggestive of long-standing dermatologic changes.

On physical examination, the patient was in fair general condition and nutritional status with vital signs within normal limits (BP 100/50 mmHg, HR 86 bpm, RR 25 breaths/min, T 36.8°C, SpO₂ 98% on room air). The skin showed erythematous and ulcerative nodules (1 cm × 2 cm) on the forearms, hands (Figure 1), feet (Figures 2, 3), and legs (Figure 4), together with scaly hyperpigmented plaques on the dorsum of the hands and irregular hypopigmented, hypoesthetic macules on the thorax and back. Mild bilateral lower-limb edema was present.

Peripheral neurological examination revealed bilateral thickening of the ulnar nerves with distal hypoesthesia of the fifth digits and decreased tactile and thermal sensitivity over the dorsum of both feet; muscle strength was 4/5 in all extremities. The remainder of the examination was unremarkable. The detailed laboratory results obtained at hospital admission are summarized in Table 1.

**Table 1:** Laboratory results at hospital admission Laboratory tests showed leukocytosis with neutrophil predominance, microcytic hypochromic anemia, thrombocytosis, and elevated acute-phase reactants (CRP and fibrinogen). Liver function tests revealed elevated transaminases, alkaline phosphatase, and gamma-glutamyl transferase, as well as hypoalbuminemia. CRP: C-reactive protein; ESR: erythrocyte sedimentation rate

Parameter	April 11	April 14	Reference range
Leukocytes (cells/mm³)	20095	20725	4500-11000
Neutrophils (cells/mm³)	16638	16248	1800-7700
Lymphocytes (cells/mm³)	1708	2632	1000-4800
Basophils (cells/mm³)	66	414	0-200
Hemoglobin (g/dL)	7.88	8.1	Male: 13.8-17.2/female: 12.1-15.1
Mean corpuscular volume (fL)	67	67	80-100
Mean corpuscular hemoglobin (pg)	22	22	27-33
Platelets (cells/mm³)	494700	627100	150000-450000
Fibrinogen (mg/dL)	946	-	200-400
CRP (mg/L)	227	287	<5
ESR (mm/h)	-	95	Male: <15/female: <20
Total bilirubin (mg/dL)	1.5	-	<1.2
Direct bilirubin (mg/dL)	0.8	-	<0.3
Aspartate aminotransferase (U/L)	96	-	10-40
Alanine aminotransferase (U/L)	117	-	7-56
Albumin (g/dL)	3.0	-	3.5-5.0
Gamma-glutamyl transferase (U/L)	380	-	8-61
Alkaline phosphatase (U/L)	536	-	44-147
Sodium (mEq/L)	133	-	135-145
Iron (µg/dL)	-	18	Male: 60-170/female: 50-160
Transferrin (mg/dL)	-	125	200-360
Ferritin (ng/mL)	-	>1000	Male: 30-400/female: 13–150
Urea (mg/dL)	-	9	15-45
Creatinine (mg/dL)	-	0.59	Male: 0.74-1.35/female: 0.59-1.04
Human immunodeficiency virus test	Negative	-	Negative
Rapid plasma reagin test (syphilis)	Positive	-	Negative

**Figure 5:**
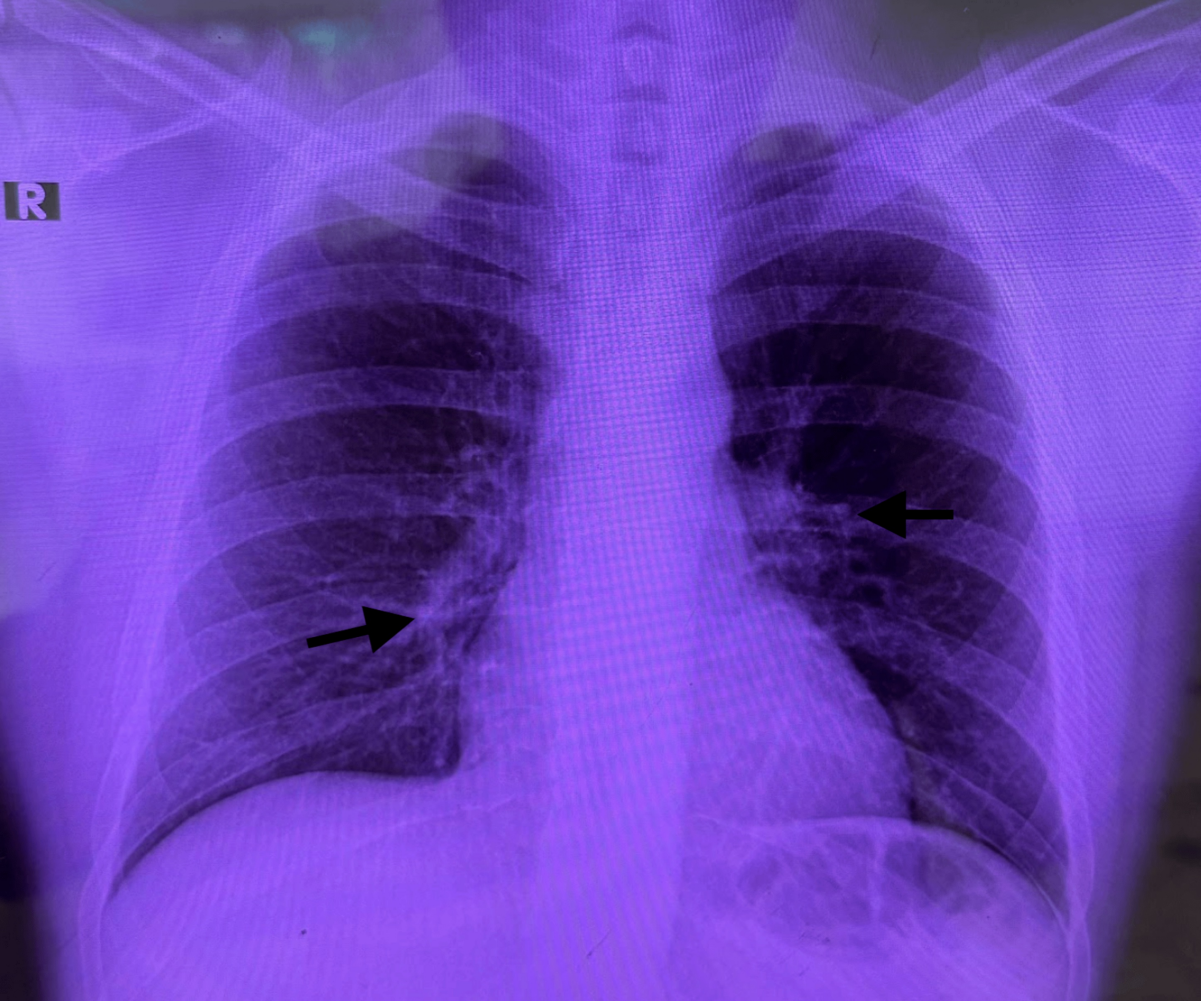
Chest radiograph Chest radiography showed well-expanded lung fields with mildly increased bronchovascular markings (arrows), a preserved alveolar-interstitial pattern, and no evidence of focal consolidation or pleural effusion. R: right

On the chest radiograph (Figure 5), well-expanded lung fields were observed, with a preserved alveolar-interstitial pattern and no evidence of pulmonary involvement.

Skin-smear microscopy for leprosy was performed using dermal smears obtained from the earlobes, elbows, and knees, following the national leprosy surveillance protocol. The bacteriological index (BI) and morphological index (MI) were determined for each site. Results showed higher bacterial loads in the earlobes and right knee, with BI values of 2+ and corresponding MI ranging from 20% to 85%, while the elbows were negative for bacilli. These findings suggest a localized bacillary distribution consistent with multibacillary leprosy (Table 2).

**Table 2:** BI and MI by sampling site Dermal smears were obtained from the earlobes, elbows, and knees according to the national leprosy surveillance protocol. BI: bacteriological index; MI: morphological index; RE: right earlobe; LE: left earlobe; REl: right elbow; LEl: left elbow; RK: right knee; LK: left knee

Sampling site	BI	MI
RE	2+	74%-85%
LE	1+	2%
REl	0	0
LEl	0	0
RK	1+	20-46%
LK	1+	2-34%

During hospitalization, he remained hemodynamically stable without respiratory or central neurological compromise. Multibacillary MDT was initiated, consisting of rifampicin 600 mg monthly (supervised), clofazimine 300 mg monthly plus 50 mg daily, and dapsone 100 mg daily. Supportive measures included symptomatic therapy, education on limb self-care, and preventive physiotherapy to avoid contractures and neuropathic ulcers.

**Figure 6:**
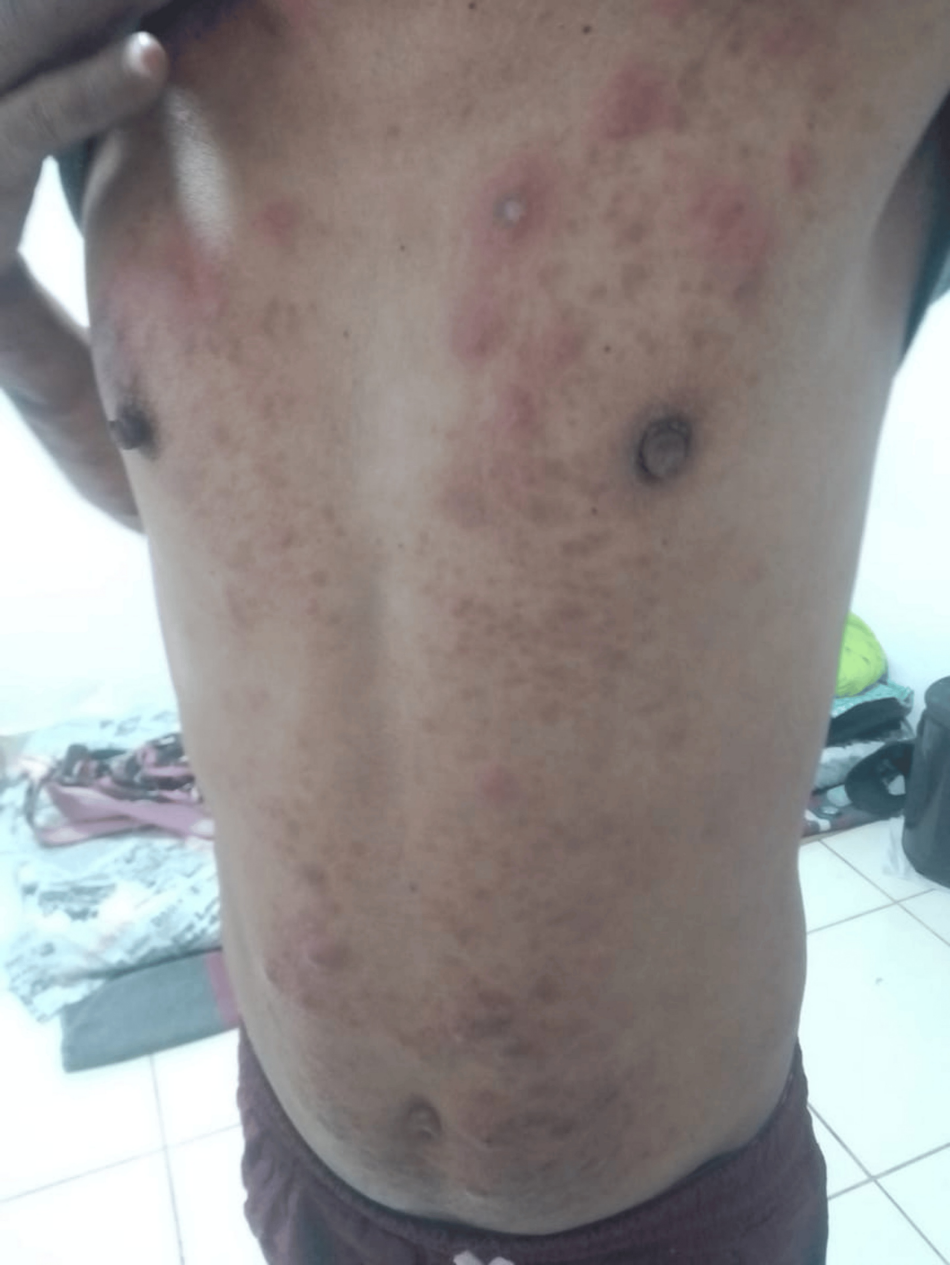
Nodules on the trunk Multiple erythematous nodules and infiltrated plaques diffusely distributed over the anterior chest and upper abdomen, some confluent, tender to palpation, and violaceous-red in color. Residual hyperpigmented macules from previous lesions are visible. No signs of active ulceration or purulent discharge are observed.

Over the following weeks, the patient demonstrated progressive improvement in skin lesions, decreased limb edema, and partial recovery of distal sensation, without drug-related adverse effects. However, in the third month of treatment, he developed painful erythematous nodules on the trunk and extremities (Figure 6), consistent with a type 2 leprosy reaction (ENL).

Bacterial coinfection and treatment interruption were excluded. Thalidomide 100 mg daily was initiated, producing marked clinical improvement within days, with resolution of erythema and pain. MDT was continued without interruption. During six months of follow-up in the Internal Medicine and Infectious Diseases clinics, no further type 2 reactions or significant adverse effects occurred. At the final evaluation, cutaneous lesions had nearly resolved, and peripheral sensory function had markedly improved, with no evidence of residual neurological disability.

## Discussion

Leprosy remains a significant public health concern and is among the 20 neglected tropical diseases recognized by the World Health Organization (WHO) [5]. Despite advances in control and elimination, active transmission persists in parts of Latin America, particularly in Venezuela and Peru, although with different magnitudes. In Venezuela, the Pan American Health Organization (PAHO) reported 338 new cases of leprosy in 2023, whereas Peru reported 26 new adult cases during the same year, with a national prevalence below one per 10000 inhabitants [6,7]. Human mobility from endemic to low-endemic regions has become increasingly relevant in the epidemiology of leprosy, facilitating the detection of imported and occasionally autochthonous cases. Similar phenomena have been reported in low-endemic countries such as the United States and Spain [8,9].

This case is noteworthy for its atypical and rapidly progressive presentation. The patient initially developed constitutional and respiratory symptoms that prompted emergency evaluation; however, radiographic studies revealed no pulmonary involvement. Although seemingly isolated, this finding highlights the diagnostic challenge of leprosy in settings where it is nearly eradicated and underscores the importance of clinical suspicion when nonspecific systemic symptoms are present. The subsequent identification of cutaneous lesions and hypoesthesia guided the diagnosis of *M. leprae* infection in its multibacillary form, confirmed by clinical and microbiological criteria.

To contextualize the atypical nature of this presentation, it is important to consider the classical clinical spectrum of leprosy. Leprosy exhibits a wide range of manifestations largely determined by the host immune response, classically ranging from tuberculoid to lepromatous disease and operationally categorized as paucibacillary or multibacillary. According to the World Health Organization, the most common clinical presentation includes hypopigmented or erythematous skin lesions accompanied by sensory loss and peripheral nerve involvement, features that often facilitate diagnosis in endemic areas [10]. Multibacillary leprosy is characterized by a higher bacillary burden, more diffuse cutaneous involvement, and an increased risk of systemic manifestations and immunological reactions. In contrast, atypical presentations such as severe reactional states, including ENL and the Lucio phenomenon, may present with ulcerative, necrotic, or vesiculobullous lesions, obscuring early recognition [11]. The aggressive cutaneous findings observed in this patient, together with systemic symptoms preceding classical dermatological signs, reflect these uncommon expressions and help explain the diagnostic complexity encountered in this case.

Given the patient’s origin and the low national prevalence of leprosy in Peru, it was hypothesized that infection occurred in his home country over five years earlier, remaining latent until recent reactivation. Laboratory results (Table 1) showed microcytic hypochromic anemia with elevated ferritin, low transferrin, and markedly increased C-reactive protein (CRP) and erythrocyte sedimentation rate (ESR), a profile consistent with anemia of chronic disease. This pattern reflects an interleukin-6-mediated immune response that enhances ferritin synthesis while suppressing hepatic transferrin production, resulting in functional hypoferremia typical of chronic infections [12]. These findings support the hypothesis of a reactivated latent infection consistent with the inflammatory pathophysiology of multibacillary leprosy. The markedly elevated ferritin and CRP levels may also reflect the intense systemic inflammatory response associated with a high bacillary burden in multibacillary disease. This inflammatory milieu has been described as a hallmark of the lepromatous pole and a predisposing factor for the development of type 2 reactions, such as ENL. Concomitantly, the initially reactive rapid plasma reagin (RPR) result was interpreted as a biological false positive rather than evidence of syphilis coinfection, given the negative confirmatory treponemal testing. Such false-positive nontreponemal reactions have also been reported in lepromatous leprosy and are attributed to immune dysregulation and circulating antiphospholipid antibodies [13,14]. The persistence of inflammatory markers further suggests endogenous reactivation rather than recent exposure, a mechanism rarely documented in low-endemic settings but possibly linked to prolonged intracellular persistence of the bacillus and individual immune modulation.

Diagnosis is based on the WHO cardinal signs: (i) sensory loss in hypopigmented lesions, (ii) thickened peripheral nerves with functional deficit, and (iii) presence of acid-fast bacilli in smears or biopsies [5]. All criteria were fulfilled in this patient (Table 2), who exhibited sensory loss, palpable nerve enlargement, and positive bacilloscopy.

Polymerase chain reaction (PCR) assays offer greater sensitivity than ELISA-based serologic tests but remain limited in clinical availability. Recent reviews confirm their diagnostic value, particularly in early paucibacillary and multibacillary forms [15,16]. Therefore, clinical recognition remains the cornerstone of diagnosis in resource-limited settings.

During therapy, the patient developed a type 2 leprosy reaction, or ENL, in the third month of treatment, manifesting as painful erythematous nodules and systemic inflammation. This reaction represents a type III hypersensitivity process mediated by immune complexes and typically occurs early during multibacillary therapy with rifampicin, clofazimine, and dapsone [13,14]. Prompt recognition and management with corticosteroids or thalidomide are essential to prevent neuropathy and permanent disability, both of which were effectively addressed in this case.

The WHO-recommended MDT, six months for paucibacillary and 12 months for multibacillary disease, remains the standard of care [5,14]. MDT effectively reduces bacterial resistance and relapse, though immune reactions continue to contribute substantially to morbidity and require close follow-up.

In the current migratory context, human mobility from countries with residual leprosy activity, such as Venezuela, to low-endemic regions including Colombia, Ecuador, Peru, and North America, poses an ongoing risk for case reintroduction and latent reactivation [17]. This underscores the need for sustained epidemiological surveillance, cross-border collaboration, and heightened clinical awareness of atypical or systemic presentations of leprosy, even in regions where the disease is considered eliminated.

## Conclusions

Leprosy remains a clinically relevant disease that requires continuous vigilance, particularly in migratory contexts and in patients with atypical presentations. As illustrated in this case, Hansen’s disease may manifest in the absence of recognized risk factors and with unusual systemic features, including respiratory symptoms without radiological abnormalities, potentially leading to diagnostic delay. In regions with high immigration, clinicians must therefore maintain a high index of suspicion for latent infection with subsequent reactivation. Furthermore, the coexistence of systemic inflammatory abnormalities and misleading serological findings may obscure early recognition, reinforcing the need to integrate laboratory data with dermatological and neurological examination. Physicians should also remain alert to the development of type 2 leprosy reactions (ENL) during the initial phase of MDT, given their significant morbidity. Early diagnosis and timely treatment remain essential to prevent irreversible nerve damage and deformities. Future clinical research is warranted to optimize early diagnostic strategies and improve comprehensive medical management of this still-neglected disease.
